# Evolving Attitudes to Ukrainian and Russian Minorities in Czechia During the Russian Invasion of Ukraine: Democrats Stay the Course

**DOI:** 10.1002/ijop.70155

**Published:** 2026-02-05

**Authors:** Martina Klicperova‐Baker, Petr Kveton, Martin Jelinek, Vít Chlad

**Affiliations:** ^1^ Institute of Psychology, Czech Academy of Sciences Brno Czech Republic

**Keywords:** attitudes, COVID‐19, Czech Republic, disinformation, minorities, Russia, Ukraine

## Abstract

This panel study examines changes in attitudes towards Ukrainian and Russian minorities in the Czech Republic and their links to disinformation beliefs and democratic commitment. The data were obtained from 490 respondents in a Czech quota sample (age 18–69; *M* = 46.09, SD = 13.40; 45.7% women). Between 2022 and 2025, mean favorability towards both groups declined: evaluations of Ukrainians shifted from slightly positive to slightly negative, while evaluations of Russians dropped from slightly to markedly negative. A repeated measures ANOVA showed that these changes were moderated by respondents' belief in disinformation and democratic orientation. Respondents resilient to disinformation and committed to democracy (‘Rational Pro‐Ukrainians’) maintained positive attitudes towards Ukrainians and showed only a medium further decline in already negative attitudes towards Russians. In contrast, respondents vulnerable to disinformation and less democratically oriented (‘Generally Disinformed’ and ‘Pro‐Russians’) displayed a sharp decline in attitudes towards Ukrainians—to strongly negative, polarising levels—while their views of Russians deteriorated only slightly. The results suggest that pro‐democratic individuals remained guided by empathy, humanism and in‐group solidarity (Social Identity Theory), whereas disinformed non‐democrats adopted out‐group, threat‐based perceptions (Realistic Threat Theory). Overall, rationality and democratic commitment appear to buffer against disinformation and polarisation, sustaining solidarity with democratic allies.

## 1 Introduction

The ideal of equality—judging people without prejudice—has become a humanistic norm. Yet, our cognitive processes rely on concrete experience and probabilistic shortcuts; conversely, we personify our social identity, typically in terms of ethnicity, race, gender, etc., to others. That inevitably undermines impartial treatment. Ethnicities are viewed with varying degrees of favorability across societies and historical periods. Empirical research indicates that with the passage of time, relations between ethnic groups improve, but interethnic prejudice persists. Interethnic relations are tested with each new migration wave, whether driven by war, climate change or political transformation.

### 1.1 Recent Waves of Immigration to Europe

Since the turn of the millennium, Europe has experienced several major immigration waves. The first followed the wars in former Yugoslavia. The next was driven by EU enlargement in 2004 and 2007, when many citizens—over a million Poles in the UK alone—moved westward for better economic opportunities. A decade later, tens of thousands of Arabs disillusioned by the failed Arab Spring migrated to the EU, foreshadowing the 2015–2016 refugee crisis, when about 2.5 million people from Syria and other unstable regions sought asylum in Europe. An even larger but very different wave came in 2022, when more than 5 million Ukrainians—mostly mothers with children—fled the Russian invasion.

Early migration waves were met with optimism. Western Europe's aging populations welcomed newcomers as both a moral duty and an economic benefit. Germany, the main destination for Syrian refugees, embraced a ‘welcome culture’ epitomised by the German Chancellor Angela Merkel's motto *Wir schaffen das* (‘We can manage’). However, the 2015–2016 New Year's Eve assaults in Cologne marked a turning point in public discourse (Wigger et al. 2022). Leading politicians declared multiculturalism a failure (BBC 2010) and public opinion shifted towards anti‐immigration stances and support of extremist parties. Similar voter realignments appeared in the 2016 Brexit referendum and Donald Trump's 2016 and 2024 victories (Goodwin and Milazzo 2017; Hooghe and Dassonneville 2018).

The Ukrainian refugee wave differed sharply from the preceding ones: refugees were culturally close to host nations and non‐threatening (women and children, as men were required to stay). Importantly, its cause was morally unambiguous—a brutal invasion marked by civilian killings, torture and the kidnapping of children. This clarity and cultural closeness prompted unprecedented solidarity, with citizens personally driving to the border for refugees and hosting them in their homes—an enthusiasm that even drew some academic criticism (e.g., Chowdhury 2024).

### 1.2 Ethnic Minorities in Czechia

Prior to World War II, substantial German, Jewish and Roma populations lived in the Czech lands. The Nazi occupation, the Holocaust and the postwar expulsion of Germans rendered the country comparatively ethnically homogeneous between 1945 and 1989. In contrast, foreign nationals in Czechia now constitute almost 10% of the 10,900,550 inhabitants (Ministry of the Interior 2024). The most numerous are *Ukrainians*. Just a few years ago, their numbers hovered around 150,500—mostly men on working visas. Following the full‐scale invasion of their homeland by Russia, a mighty refugee wave of mothers with children increased the Ukrainian minority to 574,500. While Poland accepted most refugees, the Czech Republic and Estonia sheltered the highest numbers per capita: 4.1% (Statista 2022).

The second‐largest minority consists of 119,000 *Slovaks*—mostly citizens of former Czechoslovakia, which peacefully divided in 1993 into the Czech and Slovak Republics—many moved to Czechia, attracted by its liberal environment or better economic prospects (Czech Statistical Office 2025). The third‐largest group, numbering nearly 68,000, is the *Vietnamese*. Their presence dates to the pre‐1989 communist era; historical ties were revitalised after the democratic revolution of 1989 opened borders and markets. Approximately 41,000 *Russians* are the fourth‐largest minority; many are affluent, highly educated members of the middle or upper classes.

### 1.3 Favorability of Ukrainian and Russian Minorities

Attitudes towards minorities in the Czech Republic reflect both current events and historical experience. Older Czechs who lived under communism were required to learn Russian—just as previous generations had been forced to learn German during the Nazi occupation. Communist propaganda reframed the 1968 Warsaw Pact invasion as an act of ‘brotherly assistance’. Despite decades of communist disinformation, many Czechs today see clear parallels between Putin's aggression in Ukraine and earlier totalitarian invasions of Czechoslovakia by Nazi Germany in 1938 and by the Soviet Union in 1968. This helps to explain strong Czech empathy for Ukrainians at least at the onset of the Russian invasion.

Moreover, recent events have also deepened Czech distrust of Russians, although most Russian individuals are seen to bear no responsibility for Kremlin policies. In any case, Russia labelled the Czech Republic an ‘unfriendly state’ in 2021 (TASS 2021). Russian operatives or volunteers hired by them have been implicated in hybrid warfare and sabotage actions across Europe (Barnes 2024). They include detonations of a Czech ammunition depot at Vrbětice in 2014, destruction of railway tracks in Eastern Poland in 2025, a failed arson attempt at a Prague bus depot in 2024, and many others.

Still, many Czechs perceive more similarities than differences between Russians and Ukrainians, whether due to their shared past within the Soviet Union, memories of Russian and Ukrainian organised crime mainly in the 1990s, or due to their similar physical appearance or linguistic proximity. For example, when both speak Russian or when they attempt to speak Czech, native Czech speakers are unable to distinguish between Russian and Ukrainian accents. Consequently, the two nations are sometimes wrongly perceived as being essentially the same. Prior to the full Russian invasion of Ukraine, Czech positive attitudes towards Russia and Ukraine were comparable, at 24% and 26%, respectively. However, following the unprovoked brutal invasion, public favorability towards Russia dropped to a mere 12%, while views of Ukraine improved to 37% according to STEM (2022).

### 1.4 Attitudes Towards Minorities in Critical Times: Empirical Evidence and Theoretical Framework

Intergroup attitudes emerge from a dynamic interplay of factors: Emotions—particularly negative ones—play a central role in shaping them (Haidt 2001; Baumeister et al. 2001). Cognitive schemata moulded by the flow of information and, increasingly, by disinformation, further influence these perceptions (Vosoughi et al. 2018). Times of crisis tend to intensify group competition and polarisation (Stephan and Stephan 2000; Tucker et al. 2018) or, conversely, foster solidarity and mutual understanding (Pettigrew and Tropp 2006).

Longitudinal research demonstrates the plasticity of attitudes towards minorities, with major societal disruptions often acting as catalysts for change. Such shifts depend on perceived threat and on the capacity for empathy towards out‐groups. These processes have even been observed among adolescents. For instance, Miklikowska (2018) documented developmental changes in intergroup attitudes, while Bobba et al. (2024) found that newspaper—but not Twitter—coverage of the Russia–Ukraine war increased empathy towards Ukrainians among Italian adolescents.

Elsewhere, de Rooij et al. (2015) found that the longitudinal effects of the 2011 English riots manifested primarily by fluctuations in attitudes towards Muslims, Black British and Eastern Europeans; they also observed a return of perceived threat and prejudice to pre‐riot levels within a year. In Germany, Czymara and Schmidt‐Catran (2017) showed that public reactions to the 2015 refugee crisis and New Year's Eve assaults in Cologne varied by context and group, where the public distinguished between refugees and immigrants. Altogether, these studies illustrate that crises do not shape attitudes towards minorities in a unidirectional way. Rather, the direction of attitudinal change depends on how perceived threats and empathy are framed and experienced.

These findings accord with psychological theories explaining benevolent or hostile reactions to minorities where fear—rational or symbolic—always plays a key role. For example, Realistic Threat Theory (RTT, Campbell 1965) links prejudice to competition over resources, while the more general Integrated Threat Theory (Stephan and Stephan 2000) extends prejudice to the cultural and identity domains. In contrast, Terror Management Theory (Greenberg 2012) connects prejudice and nationalism to existential anxiety. Finally, classic concepts such as the frustration–aggression hypothesis link prejudice with scapegoating (Dollard et al. 1939), and relative deprivation theory (Stouffer et al. 1949) attributes hostility to experienced frustration.

Social Identity Theory (SIT; Tajfel and Turner 1979) highlights in‐group favouritism, which may foster either exclusion or inclusion of minorities depending on perceived commonalities. One such commonality may be a compatible worldview (Brandt and Crawford 2020). Alternatively, Allport's (1954) intergroup contact hypothesis posits that direct intergroup contact reduces prejudice, while the empathy–altruism hypothesis of Batson et al. (2002) links empathic concern to prosocial action, provided distress does not override compassion (Eisenberg and Fabes 1990). Value‐based approaches, including Schwartz's (2012) theory of basic values and Haidt's (2024) moral foundations theory, also make predictions about majority feelings towards minorities. Finally, psychology and political theory converge on the Kantian idea of ‘democratic peace’, grounded in benevolence, civility and conflict resolution (e.g., Feierabend and Klicperová‐Baker 2015). For this study, RTT and SIT, complemented by the theoretical frameworks of Schwartz and Haidt, and the psychological theory of democratic peace, are apposite.

### 1.5 Knowledge Gap

Although attitudes towards minorities are well‐studied, considerably less is known about attitudes towards minorities originating from opposing sides of an armed conflict. We are not aware of any *longitudinal panel study* that has examined how such attitudes evolve. In this respect, panel surveys are important in measuring true individual‐level change: something not possible with repeated cross‐section surveys (cf. Ceobanu and Escandell 2010).

Moreover, while public sympathy for refugees often declines, it remains unclear to what degree this trend reflects *hosts' vulnerability to disinformation*—particularly within the context of Russian hybrid warfare or weak democratic commitment. This panel study addresses these gaps in the literature by (1) tracking changes and polarisation in attitudes towards minorities from war‐affected nations and (2) testing how disinformation susceptibility and democratic values moderate these trends.

### 1.6 The Current Study

This study draws on a 14‐wave longitudinal panel conducted in the Czech Republic from 2020 to 2025. The panel was launched at the onset of the COVID‐19 pandemic and initially focused on health behaviours, democratic spirit and susceptibility to disinformation. From the broader set of available measurements, only those waves containing data relevant to the research question were selected. The present article focuses on attitudes towards minorities, which were assessed in five waves conducted at non‐fixed intervals (Waves 4, 7, 10, 11 and 14). The original wave numbering was retained to facilitate cross‐referencing with other studies based on the same panel.

To examine how disinformation exposure relates to intergroup attitudes, we employed the *Disinformation Typology*. It is a classification of psychological susceptibility to disinformation developed in a companion study, ‘Susceptibility to Disinformation: A Data‐Driven Typology Based on COVID‐19 Hoaxes and Pro‐Russian Propaganda’ (Klicperova‐Baker et al. 2026). This typology, derived from the same longitudinal dataset, classifies respondents into five types by their vulnerability to disinformation, as shown in Table 1.

**TABLE 1:** Disinformation typology derived from vulnerability to medical and political disinformation.

**Generally Disinformed (8.6%, *N* = 42):** vulnerable to both COVID‐19 hoaxes and pro‐Russian political propaganda. Demography: lower education, prevalence of women (2:1), average age (46). *Democratic commitment:* Low (33.3% consider living in a democratically governed country extremely important).
**Pro‐Russians (15.1%, *N* = 74):** vulnerable to pro‐Russian propaganda, reject Russian guilt/responsibility for the war, and express economic resentment towards Ukrainians. Only mildly susceptible to COVID‐19 disinformation. Demography: the oldest type (51), 55% men, and relatively low‐educated (46% basic, 40.5% secondary and 13.5% tertiary education). *Democratic commitment:* Low (40.5% consider living in a democratically governed country extremely important)
**No Strong Opinion (47.8%, *N* = 234):** the largest and most neutral type, marked by a lack of strong views—possibly due to disinterest or reluctance. Demography: average in age (46), gender (50:50) and education (37% basic, 39% secondary, 24% tertiary). *Democratic commitment*: Low (37.2% consider living in a democratically governed country extremely important)
**Anti‐Russians (6.7%, *N* = 33):** strongly reject Russian propaganda and firmly attribute war guilt/responsibility to most Russians; moderately reject both economic resentment towards Ukrainians and COVID‐19 disinformation. Demography: older type (50), more men (2:1), even distribution of educational levels (36% basic, 30% secondary, 33% tertiary). *Democratic commitment:* High (66.7% consider living in a democratically governed country extremely important)
**Rational Pro‐Ukrainians (21.8%, *N* = 107):** strongly reject disinformation (both COVID‐19 and Russian propaganda) and most strongly reject economic resentment toward Ukrainians; ambivalence regarding the war guilt/responsibility of most Russians likely reflects liberal opposition to collective blame. Demography: the youngest type (42), prevalence of men (2:1) and the highest education (almost half tertiary). *Democratic commitment:* Very high (83.2% consider living a democratically governed country extremely important)

*Note:* Based on *Susceptibility to Disinformation: A Data‐Driven Typology Based on COVID‐19 Hoaxes and Pro‐Russian Propaganda* by Klicperova‐Baker et al. 2026, International Journal of Psychology. The labels (e.g., *Pro‐Russians* and *Anti‐Russians* in the above typology) are interpretations of Latent Profile Analysis (LPA) of susceptibility to COVID‐19 hoaxes and to Russian political propaganda. The disinformation typology was based on the same respondents as the present study but relied on a different set of measurement items that did not include attitudes towards minorities, which are central to the current analysis. The supplementary index of democratic commitment represents the percentage of respondents within each type who rated living in a democratic country as ‘extremely important, the highest point of the ten‐point scale’.

The primary aims of this study are:

- To track changes in attitudes towards Ukrainian and Russian minorities in the Czech Republic from 2022 to 2025;
- To analyse the differentiation and polarisation processes of these attitudes using the disinformation typology introduced in our previous study (Klicperova‐Baker et al. 2026);
- To validate these findings with two supplementary questions, that is, perceptions of Ukrainians as an economic asset or burden for the Czech Republic and spontaneous verbal associations with the word ‘Ukraine’.

Based on these aims, we posit the following three expectations. First, a decline in Czech likability towards both the Russian and Ukrainian minorities between February 2022 and January 2025. Second, an increase in polarisation among Czechs—particularly in attitudes towards the Ukrainian minority—across disinformation types. Third, a consistent and logical correspondence between these results and the supplementary questions dealing with economic perceptions and verbal associations.

## 2 Method

### 2.1 Participants and Data Collection

We used a longitudinal Czech national panel survey with repeated measures, quota‐sampled by key demographics (gender, age, education, settlement size and region). Attitudes towards minorities were measured in Waves 4 (February 4–15, 2022), 7 (May 24–June 16, 2022), 10 (February 16–March 1, 2023), 11 (November 3–16, 2023) and 14 (December 4–17, 2024 and January 7–17, 2025). The numbering of waves corresponds to the full 14‐wave project to ensure consistency across all analyses derived from it. The number of participants per wave ranged from 863 to 1192, with retention rates never falling below 77% of the original sample (Wave 4 = 93.6%, Wave 7 = 100%, Wave 10 = 91.4%, Wave 11 = 85.1% and Wave 14 = 77.1%). Data collection was conducted by Median, an established Czech polling agency, using computer‐assisted web interviewing (CAWI). Respondents received a modest financial incentive (equivalent to approximately $1–2 USD) for each completed wave.

### 2.2 Measures

*Attitudes towards ethnic minorities* were assessed with the question ‘Using this scale, how would you describe your relationship to the population groups living in the territory of the Czech Republic?’ followed by a randomly rotated list of ethnic groups (Table 2) and a Likert response scale ranging from 1 = *very likable* to 5 = *very unlikable*. The first measurement took place just weeks before the 2022 Russian full‐scale invasion of Ukraine, with replications in months 3, 12, 20 and 34 after the invasion. To simplify interpretation, we reversed the scores before analysis so that a higher value reflected greater likability.

**TABLE 2:** Average likability (scale 1 to 5) of various ethnic groups in the Czech Republic before the 2022 invasion.

Ethnic group	Likability: *M* (SD)
1. Czechs	4.15 (0.89)
2. Slovaks (the second most populous minority)	4.06 (0.89)
3. Vietnamese (the third most populous minority)	3.60 (0.87)
4. Greeks	3.48 (0.78)
5. Jews	3.40 (0.91)
6. Poles	3.33 (0.83)
7. Germans	3.24 (0.92)
8. Hungarians	3.20 (0.82)
9. Ukrainians (the most populous minority)	3.12 (0.94)
10. Bulgarians	3.00 (0.81)
11. Serbs	3.00 (0.83)
12. Chinese	2.94 (0.87)
13. Romanians	2.68 (0.87)
14. Russians	2.67 (1.03)
15. Albanians	2.42 (0.89)
16. Arabs	2.17 (0.94)
17. Roma (very numerous by unofficial count)	2.02 (0.92)

*Note:* The likability of individual nationalities differed significantly (*F*(16, 7328) = 280.11, *p* < 0.01). Based on Tukey's post hoc test for the within‐subject effect within the general linear model, statistically significant differences (*p* < 0.05) were found for all pairs of nationalities except for the following pairs: 1–2; 3–4; 4–5; 4–6; 5–6; 5–7; 5–8; 6–7; 7–8; 7–9; 8–9; 9–10; 9–11; 10–11; 10–12; 11–12; 13–14; 16–17.

Respondents' *free associations with the word* ‘Ukraine’ were measured with an open‐ended probe administered in Wave 10, on the first anniversary of the 2022 Russian invasion. The instruction for this item was: ‘One of the most frequently used words today is “Ukraine”. What does Ukraine mean to you? What does it symbolize? Please write three answers that first come to your mind’.

Finally, there was an item that explored Czechs' *perceptions of the economic impact of Ukrainian refugees on the state budget*. As part of Wave 14, respondents were asked, ‘What do you think is closest to the truth?’ The response options ranged from 1 = *Refugees from Ukraine still represent a net cost to the state* to 5 = *Refugees from Ukraine already contribute more to the state than is the cost of their support*. According to the Czech Ministry of Labor and Social Affairs (2025) data, response option ‘5’ was the correct answer.

### 2.3 Analytical Procedure

1. Review Czechs' attitudes towards multiple minority groups in Wave 4 of the panel survey.
2. Analyse the evolution of Czech attitudes towards the Russian minority in Waves 4, 7, 10, 11 and 14. Initially, examine the ratings from the full sample. Then repeat the analysis with subgroups characterised by different levels of disinformation susceptibility based on the Disinformation Typology developed in a previous study (see Table 1). Classify respondents and compare attitudes across disinformation types using repeated measures ANOVA to assess differences in favourability across waves.
3. Analyse the evolution of Czech attitudes towards the Ukrainian minority using the same procedure described in the previous step.
4. Augment the analysis of the disinformation profiles using two additional attitudinal indicators: (a) perception of Ukrainian refugees being an economic asset or a burden for the Czech Republic and (b) verbal associations for the word ‘Ukraine’.

## 3 Results

### 3.1 Measurement of Attitudes Towards Multiple Minority Groups in Wave 4

Table 2 presents favorability ratings of ethnic groups (data from Wave 4), conducted in February 2022, 11 to 21 days prior to the full‐scale Russian invasion of Ukraine. The results indicate that Czech respondents differentiated among foreign groups in terms of favorability. Specifically, most foreigners were rated positively, particularly the largest minorities: Ukrainians, Slovaks and Vietnamese. An exception was the domestic Roma/Gypsies, who were generally rated unfavourably. Ukrainians were rated more favorably than Russians. The average Czech self‐rating was 4.15, only slightly higher than the 4.06 score assigned to Slovaks.

### 3.2 Attitudes Towards the Russian Minority—A Longitudinal View

**FIGURE 1:**
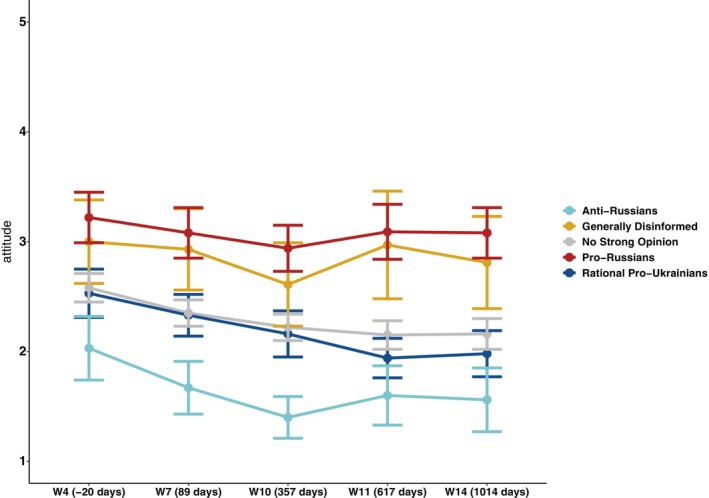
Likability of the Russian minority in Czechia by disinformation‐type membership, shortly before and after the full‐scale Russian invasion of Ukraine in 2022. The vertical axis shows mean likability on a 5‐point Likert scale (1 = *Very negative*, 5 = *Very positive*) with 95% confidence intervals. On the horizontal (time) axis, the numbers in parentheses indicate the number of days since the start of the full Russian invasion (February 24, 2022) in terms of the beginning of data collection for each wave.

*Average ratings for the full sample* are presented in the top half of the first column (‘Total’) of Table 3. Over time, the mean full sample favorability ratings towards Russians *declined by 0.40 points* from mildly negative (*M* = 2.67, Wave 4) to markedly negative (*M* = 2.27, Wave 14). Examining *average ratings of the Russian minority by disinformation types*, Table 3 and Figure 1 show that at the onset of our measurement (Wave 4), polarisation was already emerging between Czech respondents who would later be categorised into five disinformation types based on their disinformation susceptibility. The future *Anti‐Russian* type expressed particularly negative attitudes (*M* = 2.03); attitudes of the future *Pro‐Russian* type were mildly positive (*M* = 3.22); the *difference between them in Wave 4 amounted to 1.19 points*.

**TABLE 3:** Evolution of Czech public attitudes to Russian and Ukrainian minorities.

Group size (*n*)	Total	Generally disinformed	Pro‐Russians	No strong opinion	Anti‐Russians	Rational pro‐Ukrainians
	490	42	74	234	33	107
Towards the Russian minority						
Wave 4	2.67 (1.03)	3.00 (1.20)	3.22 (0.95)	2.58 (0.95)	2.03 (0.80)	2.53 (1.07)
Wave 7	2.46 (1.05)	2.93 (1.18)	3.08 (0.98)	2.35 (0.99)	1.67 (0.69)	2.33 (1.00)
Wave 10	2.30 (1.01)	2.61 (1.13)	2.94 (0.87)	2.22 (0.94)	1.40 (0.50)	2.16 (1.02)
Wave 11	2.28 (1.06)	2.97 (1.30)	3.09 (1.04)	2.15 (0.94)	1.60 (0.72)	1.94 (0.88)
Wave 14	2.27 (1.04)	2.81 (1.17)	3.08 (0.86)	2.16 (0.96)	1.56 (0.75)	1.98 (0.96)
Δ Wave 4–14	0.40	0.19	0.14	0.42	0.47	0.55
Towards the Ukrainian minority						
Wave 4	3.12 (0.94)	3.00 (1.20)	2.99 (0.88)	3.05 (0.93)	3.13 (1.02)	3.42 (0.79)
Wave 7	3.09 (0.95)	2.57 (1.09)	2.66 (0.94)	3.03 (0.89)	3.33 (0.85)	3.64 (0.74)
Wave 10	2.96 (0.97)	2.14 (0.93)	2.44 (0.94)	2.90 (0.89)	3.47 (0.73)	3.63 (0.68)
Wave 11	2.78 (1.04)	1.73 (0.91)	2.15 (0.89)	2.76 (0.95)	3.23 (0.97)	3.48 (0.81)
Wave 14	2.71 (1.04)	1.81 (0.83)	2.02 (0.88)	2.69 (0.96)	3.07 (0.92)	3.47 (0.79)
Δ Wave 4–14	0.41	1.19	0.97	0.36	0.06	−0.05

*Note:* The estimates refer to the mean and standard deviations of 5‐point Likert scales where 1 = *Very unlikable* and 5 = *Very likable*. Δ Wave 4–14 denotes the difference between Wave 4 and Wave 14 (earlier minus later). Positive values indicate a decline in likability over time.

Repeated‐measures ANOVA confirmed a further gradual worsening of attitudes towards Russians following the full invasion (*F*(4, 1682) = 27.04, *p* < 0.01); the magnitude of attitude change was conditioned by disinformation type (*F*(16, 1682) = 1.88, *p* < 0.05). All five types gradually intensified their negativity towards Russians from the beginning of our fieldwork until Wave 10 (the first anniversary of the full invasion), when negativity towards Russians culminated in three of the five types. Subsequent waves 11 and 14 showed a further decline in moderate *Rational Pro‐Ukrainians* and *No Strong Opinion*; others reverted to milder levels of negativity, mostly to values observed by May–June 2022 (Wave 7). By the end of the study period, the *difference* between largely stabilised *Anti‐Russian* and *Pro‐Russian scores in Wave 14 amounted to 1.52 points*. These data suggest mild polarisation.

### 3.3 Attitudes Towards the Ukrainian Minority—A Longitudinal View

**FIGURE 2:**
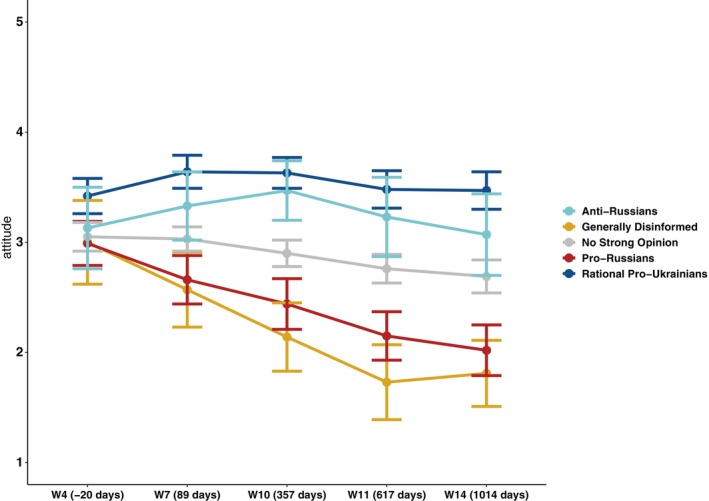
Likability of the Ukrainian minority in Czechia by disinformation‐group membership, shortly before and during the 2022 full‐scale Russian invasion of Ukraine and after a surge in refugee arrivals from Ukraine. The vertical axis shows mean likability on a 5‐point Likert scale (1 = *Very negative*, 5 = *Very positive*) with 95% confidence intervals. On the horizontal (time) axis, the numbers in parentheses indicate the number of days since the start of the full Russian invasion (February 24, 2022) in terms of the beginning of data collection for each wave.

*Average ratings for the full sample* are presented in the lower half of the first column (Total) of Table 3. Over time, mean favorability ratings towards Ukrainians *declined* from mildly positive (*M* = 3.12, Wave 4) to mildly negative (*M* = 2.71, Wave 14), that is, by the same amount as in the case of Russians—*by 0.40 points*. Turning now to the *average ratings of the Ukrainian minority by disinformation types*, Table 3 and Figure 2 show that at the onset of our panel survey fieldwork (Wave 4), there was a considerable consensus between respondents who would later be categorised into five disinformation types: *Only 0.43 points separated* the most positive rating (*M* = 3.42) of future *Rational Pro‐Ukrainians* and the lowest but still neutral rating (*M* = 2.99) of future *Pro‐Russians*. As with attitudes towards Russians, a repeated‐measures ANOVA revealed two things: (1) a significant effect of time on attitudes towards Ukrainians, *F*(4, 1682) = 37.69, *p* < 0.01, and (2) a significant time × disinformation type interaction, that is, change in favorability over time was conditioned by group membership, *F*(16, 1682) = 6.98, *p* < 0.01.

In the evolution of Czech attitudes to the Ukrainian minority, the *No Strong Opinion* type remains closest to the neutral midpoint; however, it gradually trends into becoming mildly negative (*M* = 2.69, Wave 14). Meanwhile, the *Generally Disinformed* and *Pro‐Russian* types shift even further into strongly negative territory, whereas the democratic *Anti‐Russians* and especially *Rational Pro‐Ukrainians* remain positive. By early 2025 (Wave 14), the *difference* between relatively stabilised *Anti‐Russian* and *Generally Disinformed* scores *amounted to 1.52 points*. *This finding indicates a substantial one‐sided polarisation towards the Ukrainian minority within Czech society*.

### 3.4 Perceived Economic Impact of Ukrainian Refugees on the Czech State Budget by Disinformation Types

The expectation that the five disinformation types would differ significantly in their perception of the economic impact of Ukrainian refugees was confirmed (*F*(4, 373) = 81.33, *p* < 0.001). Specifically, *Rational Pro‐Ukrainians* and *Anti‐Russians*, in line with the latest statistics from the Czech Ministry of Labor and Social Affairs (2025), predominantly viewed Ukrainian refugees as an economic asset (*M* = 4.16, SD = 1.08; *M* = 3.67, SD = 1.14, respectively, on a 5‐point scale). As expected, the *Pro‐Russian* and *Generally Disinformed* types generally viewed Ukrainian refugees wrongly as a financial burden (*M* = 1.39, SD = 0.64; *M* = 1.45, SD = 1.03, respectively). The *No Strong Opinion* type, which is closest to the scale midpoint, also leaned towards scepticism (*M* = 2.37, SD = 1.15). In sum, these findings cross‐validate the disinformation typology developed in previous work and its use in this study.

### 3.5 Free Associations to the Word ‘Ukraine’ by Disinformation Types

To bypass some limitations of self‐reported prejudice and associated social desirability effects, a free association method was used to explore Czechs' latent attitudes towards Ukraine and its people. To complement responses to quantitative scales, respondents were also given the opportunity to express their feelings qualitatively through free associations to the stimulus word ‘Ukraine’. Table 4 summarises typical responses by group membership. The *No Strong Opinion* group typically reacted with a rather impartial word (war or refugee) or by negative or compassionate emotions. In contrast, *Pro‐Ukrainian Realists* and *Anti‐Russians* primarily expressed admiration for Ukrainian heroism, while *Pro‐Russians* reacted with alleged Ukrainian crimes (typically, corruption) and also stood out by either linking Ukraine to fascism, Nazism, the name of Stepan Bandera, or ostentatiously responded that they could not come up with any response at all. Finally, the *Generally Disinformed* typically mentioned some Ukraine‐related economic grievance, a neutral response (such as war or a neutral geographic association), or failed to respond. These associations appear to further meaningfully illustrate and validate the disinformation typology.

**TABLE 4:** Typical free associations to the word ‘Ukraine’ among Czechs by group membership.

Type	Typical associations
No strong opinion	(1) War (2–3) Negative or compassionate emotions (fear, compassion, insecurity, helplessness, anger) (2–3) Refugees (4) Suffering, destruction, death
Rational pro‐Ukrainians	(1) Heroism, courage, resolution, defiance (2) War (3) Suffering, destruction, pain, victims, destroyed country (4) Help, admiration, compassion, solidarity, support
Pro‐Russians	(1) Ukrainian crimes: corruption, mafia, extortion, criminal elements (2) War (3) Fascism, Nazism, Stepan Bandera (an internationally contested historical Ukrainian figure) (4) Nothing
Generally disinformed	(1) Economic grievances (inflation, waste of money, impoverishing Czechia) (2) War (3) Economic or geographic associations (country, crops, mineral riches) (4) I do not know, nothing
Anti‐Russians	(1–2) Heroism, courage, resistance, cohesion (1–2) Suffering, death, destruction (3) War (4–5) Freedom or war politics (Russian aggression, defence of Europe)

## 4 Discussion

The observed average decline in the likability of both Russian and Ukrainian minorities over time was expected. The former could be attributed to the cumulative effects of ongoing aggression and civilian suffering; the latter to the large influx of refugees combined with the current wave of xenophobic populism (e.g., Kende and Krekó 2020). Remarkably, the average likability of Russians and Ukrainians decreased by almost identical amounts (0.41 and 0.40 points, respectively; see the Δ rows in Table 3). However, while the likability of the Russian minority declined across all groups (for obvious reasons), the decline in attitudes towards the Ukrainian minority was driven mainly by two disinformed types: *the Generally Disinformed* (1.19) and *Pro‐Russians* (0.97). It is important to note that the five disinformation types are not based on attitudes towards minorities data but on responses about mis‐ and disinformation (see the note in Table 1).

### 4.1 Disinformation and Reframing of Attitudes Towards the Ukrainian Minority

At the beginning of this study, in the spring of 2022, Czech respondents displayed a broad consensus characterised by solidarity with Ukraine. Their attitudes towards Ukrainians clustered in the mildly positive range, likely reflecting the influence of humanitarian framing. However, following the full‐scale Russian invasion, the arrival of over 400,000 refugees in Czechia and the intensification of Russian propaganda, these attitudes began to diverge: Czechs high in democratic orientation and less affected by disinformation ‘stayed the course’ in their support for Ukrainian refugees. In contrast, others, particularly the two significantly disinformed types with low democratic commitment, became increasingly unsympathetic or even hostile. They most likely underwent a shift from humanitarianism to *materialism, a threat‐based framing*.

In line with this reasoning, the pro‐democratic, well‐informed respondents—*Rational Pro‐Ukrainians* and *Anti‐Russians—*maintained the humanitarian perspective and stable sympathy. Consistent with Social Identity Theory (SIT), they tend to perceive Ukrainians as an in‐group: fellow Europeans, fellow democrats and brave fellow victims of Russian imperialism—sharing a similar worldview (Brandt and Crawford 2020; Havermans and Verkuyten 2021). They likely engaged the ‘self‐transcendent’ values of ‘universalism’ and ‘benevolence’ (Schwartz 2012; Davidov et al. 2008), together with the moral foundations of ‘care’ and ‘liberty’ (Haidt 2024). Allport's intergroup contact hypothesis also generally applies to this group. Associations expressing sympathy—such as ‘heroism’ and ‘courage’—were their typical spontaneous responses to the word ‘Ukraine’, as shown in Table 4; these respondents were also aware that refugees were no longer a burden but an asset to the Czech social system and economy.

In contrast, the *Generally Disinformed* and *Pro‐Russian* types—characterised by high susceptibility to disinformation and weaker democratic commitment—displayed a steep decline in sympathies towards Ukrainians. Economic and symbolic threat frames, reinforced by misleading narratives (e.g., ‘Ukrainians live at your expense’), gradually replaced the initial humanitarian framing. In this process, ‘refugees’ quickly became ‘threatening immigrants’ in the eyes of these respondents (cf. Czymara and Schmidt‐Catran 2017). Any of the theories of threat, frustration–aggression or relative deprivation may apply here. When individuals experience economic hardship or a sense of marginalisation, they are particularly receptive to foreign propaganda. Material concerns—inflation, financial strain and fears of national impoverishment—frequently appeared among free associations to ‘Ukraine’ by the *Generally Disinformed* group. Members of both highly disinformed types also incorrectly reported Ukrainian refugees as an economic burden rather than an asset to the Czech Republic.

Finally, the *Pro‐Russian* type appears to have one additional imprint—an ideological burden. It likely reflects the legacy of communism, the Red Army and the Soviet Union—recently reframed by Russian propaganda as the imperial defence of ‘traditional values’ against Western liberalism. Within this group, disinformation operates not merely as a catalyst but as a proximate causal factor, reinforcing preexisting ideological commitments through motivated reasoning (Kunda 1990). Characteristically, when prompted with the word ‘Ukraine’, members of this group produced associations related not to economy but to ideology.

### 4.2 Ideology and Stability in Attitudes Towards the Russian Minority

A key trend in Table 3 shows that the likability of the Russian minority declined across all groups, with the steepest decreases observed among *Rational Pro‐Ukrainians* and *Anti‐Russians*. Even respondents with *No Strong Opinions* became less in favour of them, while the *Generally Disinformed* and *Pro‐Russian* types showed only a small decline, maintaining a more positive view of Russians than others. Not surprisingly, the *Anti‐Russians* displayed the strongest antipathy, whereas the *Pro‐Russians* expressed the strongest—though still mild—sympathy. These two types are also the oldest, suggesting that personal historical experiences of the 1968 invasion, on the one hand, and ideological imprinting from the communist period of Czechoslovakia, on the other, may both still play some role in their case whether due to firsthand experiences or from narratives transmitted by their parents. In the case of attitudes towards Russia, the measured reactions of ‘democrats’ (i.e., those who agreed that living in a democracy is ‘extremely important’) suggest that they have not undergone a substantial change in their frame of reference. They appear to have learned little new about Russians during the invasion and were not measurably influenced by targeted disinformation.

### 4.3 Asymmetric Polarising Trends

In classic cases of polarisation, the political center tends to erode as the distribution of attitudes splits into two opposing camps. Such a pattern was not observed here. The largest, centrist, *No Strong Opinion group* remained moderate, although showing a slight negative shift between 2022 and 2025. This large centrist group does not appear to fragment; its attitudes towards both minorities maintained narrow confidence intervals (see Figures 1 and 2). The modest decline in attitudes towards Ukrainians may reflect war‐related fatigue as the conflict persists and temporary refugees become long‐term residents.

However, the data show asymmetric—one‐sided—polarisation in groups with firm opinions towards ideologically dissimilar groups: The pro‐democratic types reported a slight worsening of attitudes towards Russians, likely due to cumulative exposure to reports of war‐related suffering. In contrast, the types vulnerable to disinformation—*Generally Disinformed* and *Pro‐Russian*—exhibited a much sharper shift towards hostility towards the Ukrainian minority. The widening range of responses results mainly from increased negativity and radicalisation among the most disinformed respondents. Similar dynamics have been observed in other countries as well (Levitsky and Ziblatt 2018).

### 4.4 Democratic Commitment as a Protective Factor Against Disinformation

The data presented in this study suggest that democratic commitment may provide immunity to disinformation. In our sample, two pro‐democratic types appeared particularly resilient: (a) *Rational Pro‐Ukrainians*—predominantly younger, male, well‐educated and likely liberal—and (b) *Anti‐Russians*—a smaller, mostly older male group characterised by attributing collective guilt or responsibility for the invasion to Russians. Overall, self‐identified democrats—whether liberal or not—emerged as the most resistant to disinformation, unfounded hostility and social divisiveness. This is consistent with the notion that social cohesion can buffer against a conspiratorial mindset (Hartz et al. 2025). However, causal mechanisms cannot be inferred from this study's data, and our findings warrant further empirical examination.

## 5 Limitations of the Study and Generalisability

This research relied on a non‐probabilistic quota sample surveyed across multiple waves. Moreover, only about half of the original panel participants completed all relevant waves. Both the non‐random nature of the sample, the potential effects of panel attrition, and the uneven size of ideological types should therefore be considered when interpreting the findings.

Another limitation is the absence of an internal baseline. Although our data collection began before the full‐scale Russian invasion, as an anonymous reviewer cogently pointed out, that period was already marked by escalating Russian threats and hostilities. The CVVM opinion poll agency, using the same methodology prior to the COVID‐19 pandemic, reported mildly negative ratings for both groups (Russians: *M* = 2.85; Ukrainians: *M* = 2.82), which may serve as the closest external benchmark (CVVM 2023). By contrast, our first measurement—conducted 2 weeks before the invasion—already indicated a positive shift in evaluations of Ukrainians (*M* = 3.12) and a more negative evaluation of Russians (*M* = 2.67).

This study focuses on a medium‐sized Central European country with a distinctive historical trajectory shaped by the experience of Russian occupation (1968–1991) and, more recently, a large influx of Ukrainian refugees. Despite these contextual specificities, the general patterns identified here are likely to possess broader international relevance. Nevertheless, although the sample was diverse in terms of age and gender, it may not fully capture the perspectives of all demographic groups—particularly those less likely to participate in surveys or to use modern communication technologies.

## 6 Future Research Directions

The work reported in this study may be extended in three main ways. First, there is the key theme of psychological predispositions and causal mechanisms of democratic resilience. Future research should investigate which constellations of psychological predispositions foster democratic commitment and strengthen individuals' resilience to disinformation and should also aim to establish the causal dynamics linking these predispositions to both democratic engagement and resistance to disinformation. A second substantive topic relates to countering disinformation and the challenges of debiasing citizens. Since the findings implicate disinformation as a key contextual factor, future research should address the resistance of the disinformed to correction once propaganda becomes embedded within complex attitudinal structures (e.g., Carey et al. 2025; Nyhan and Reifler 2010). To this end, subsequent studies should integrate multifaceted measures—from neuropsychology to political psychology—to uncover the psychological sources of both resilience to and prevention of disinformation. Finally, there is scope for developing *cross‐national perspectives*. Fortunately, the panel data used in this study involved a heterogeneous group of participants willing to contribute to a longitudinal project that allows us to continue our observations in the Czech context. As a logical next step, it is important to move beyond a motivating case study to develop comparative methods that facilitate a generalised study of the trajectories of disinformation.

## 7 Conclusions

This study examined the evolution of Czech attitudes towards Russian and Ukrainian minorities during the ongoing Russian invasion of Ukraine. Over the observed period, mean favorability towards both groups declined. The initial surge of solidarity with Ukraine was followed by an increasing diversification of attitudes that could be predicted from respondents' disinformation profiles. Overall, attitudes towards the Ukrainian minority shifted from slightly positive to slightly negative, whereas evaluations of the Russian minority moved from mildly to markedly negative.

A repeated measures ANOVA indicated that these changes were moderated by respondents' susceptibility to disinformation and their democratic orientation. Respondents resilient to disinformation and committed to democratic values—particularly the *Rational Pro‐Ukrainians*—maintained their humanitarian and normatively grounded perspectives, and with them, positive attitudes towards Ukrainians. This group also exhibited only a moderate additional decline in their rather negative attitudes towards Russians.

In contrast, respondents susceptible to disinformation and propaganda—*Generally Disinformed* and *Pro‐Russians*—displayed a sharp decline in positive attitudes towards Ukrainians to strongly negative, hostile levels, perceiving Ukrainians as an economic (the *Generally Disinformed* type) and possibly an ideological threat (*Pro‐Russian* type). These findings indicate one‐sided radical polarisation among the most disinformed respondents. While the *Generally Disinformed* tended to view Ukrainians as an economic threat, the *Pro‐Russian* group increasingly perceived them as an ideological one. These findings point to a one‐sided, radical form of polarisation concentrated among the most disinformed Czech respondents.

In sum, the evidence presented in this study suggests that rationality and democratic commitment not only provide psychological protection against disinformation and polarisation, but also actively sustain social solidarity with democratic Ukraine and its people in the face of invasion by an aggressive non‐democratic neighbour. Taken together, these findings offer tentative support for the idea that democratically committed citizens do indeed stay the course when confronted with disinformation, polarisation, and external aggression.

## Author Contributions

**Martina Klicperova‐Baker:** conception and design; collection of data; drafting the article. **Petr Kveton:** analysis and interpretation of data; drafting the article. **Martin Jelinek:** analysis and interpretation of data. **Vít Chlad:** analysis and interpretation of data.

## Funding

This study was funded by the project Mediated Society (MEDIS: ON) No. CZ.02.01.01/00/23_025/0008713—MEDIS:ON provided by MŠMT; the NPO ‘Systemic Risk Institute’ funded by the European Union—Next Generation EU (Ministry of Education, Youth and Sports, NPO: EXCELES), LX22NPO5101; and Strategy AV21/32 Grant ‘Identities in the World of Wars and Crises’ by the Czech Academy of Sciences and a COVID grant by IOCB, Prague.

## Ethics Statement

All procedures complied with the principles of the Declaration of Helsinki (1964) and its later amendments. Respective waves of the study were approved by the Institutional Ethics Committee of the Institute of Psychology, Czech Academy of Sciences. Moreover, the opinion‐poll agency Median fully complies with applicable EU legislation (including GDPR) and the ICC/ESOMAR Code of Conduct. Only adults were eligible to participate.

## Consent

Informed consent was obtained from all individual participants included in the study and participants were free to withdraw at any time.

## Conflicts of Interest

The authors declare no conflicts of interest.
